# Biomechanics of lower limb in badminton lunge: a systematic scoping review

**DOI:** 10.7717/peerj.10300

**Published:** 2020-11-04

**Authors:** Wing-Kai Lam, Duo Wai-Chi Wong, Winson Chiu-Chun Lee

**Affiliations:** 1 Guangdong Provincial Engineering Technology Research Center for Sports Assistive Devices, Guangzhou Sport University, Guangzhou, China; 2 Department of Kinesiology, Shenyang Sport University, Shenyang, China; 3 Li Ning Sports Science Research Center, Li Ning (China) Sports Goods Company, Beijing, China; 4 Department of Biomedical Engineering, Faculty of Engineering, The Hong Kong Polytechnic University, Hong Kong, China; 5 School of Mechanical, Materials, Mechatronic & Biomedical Engineering, University of Wollongong, Wollongong, New South Wales, Australia

**Keywords:** Plantar pressure, Kinematics, Kinetics, Lunging, Footwear

## Abstract

**Background:**

Badminton is a popular sport activity in both recreational and elite levels. A lot of biomechanical studies have investigated badminton lunge, since good lunge performance may increase the chances to win the game. This review summarized the current trends, research methods, and parameters-of-interest concerning lower-extremity biomechanics in badminton lunges.

**Methodology:**

Databases including Web of Science, Cochrane Library, Scopus, and PubMed were searched from the oldest available date to September 2020. Two independent authors screened all the articles and 20 articles were eligible for further review. The reviewed articles compared the differences among playing levels, footwear designs, and lunge directions/variations, using parameters including ground reaction forces, plantar pressure distribution, kinematics, and kinetics.

**Results:**

Elite badminton players demonstrated higher impact attenuation capability, more aggressive knee and ankle strategy (higher mechanical moment), and higher medial plantar load than amateur players. Footwear modifications can influence comfort perception and movement mechanics, but it remains inconclusive regarding how these may link with lunging performance. Contradicting findings in kinematics is possibly due to the variations in lunge and instructions.

**Conclusions:**

Playing levels and shoe designs have significant effects on biomechanics in badminton lunges. Future studies can consider to use an unanticipated testing protocol and realistic movement intensity. They can study the inter-limb coordination as well as the contributions and interactions of intrinsic and extrinsic factors to injury risk. Furthermore, current findings can stimulate further research studying whether some specific footwear materials with structural design could potentially compromise impact attenuation, proprioception, and performance.

## Introduction

Badminton is recognized as the second most popular participation sport. More than 200 million participants play badminton in recreational and elite levels worldwide (Phomsoupha & Laffaye, 2015). Badminton is a high-intensity intermittent racket sport that requires a high level of technical skills, tactics, and physical capacities during training and competition (Faude et al., 2008). While footwork and lower-limb movements are particularly important in badminton games, biomechanical analysis can provide good insight into how these movements should be optimized.

Various strenuous manoeuvres such as lunging, turning, sprinting, leaping, jumping, and landing can play a critical role in badminton plays (Kuntze, Mansfield & Sellers, 2010). Lunge manoeuvre has been commonly reported in the literature, as it accounts for over 15% of the game time. There are averages of 52.2 moves of half lunges and 46.1 moves of forward lunges in a match, and more than half of these lunges involve diagonal movements (Valldecabres et al., 2020). Mastering lunge can potentially improve performance and reduce injury potential in badminton (Kuntze, Mansfield & Sellers, 2010; Valldecabres et al., 2020). During lunges, players need to maintain a high level of core and knee dynamic stability to accommodate the rapid changes of body positions, which is commonly indicated by substantial alterations to the center of mass displacement and center of pressure excursion. Stable and rapid movements allow the upper limbs to be in the best position (Huang et al., 2014) to hit the shuttle or to produce a counter-attack shot. Professional players should lunge and reach the shuttlecock at the best position quickly and maintain balance with skillful and stable footwork (Chin et al., 1995; Hong et al., 2014). However, the demanding footwork could also result in high injury risks at knee and ankle joints, with incidence rates from 63% up to 92% (Herbaut, Delannoy & Foissac, 2018). Players could experience impact load as high as 2.5 times of the body weight and require sufficiently high muscle activities to stabilize lower extremities joint during a lunge (Phomsoupha & Laffaye, 2015). This loading could lead to muscle fatigue, discomfort, pain, and injuries (Boesen et al., 2006; Hu et al., 2015).

There was a paradigm shift of research focus on badminton lunge, from strength qualities to movement mechanics. Studies in the past decades predominantly focused on the determinants of lunge performance using isolated tests on strength qualities (Cronin, McNair & Marshall, 2003; Thijs et al., 2007). The advancement of the motion capture systems and other instruments stimulated kinematic studies on the upper limb and racket mechanics (Kwan et al., 2011; Kwan et al., 2010). In this study, we endeavored to investigate the dynamics of the lunge manoeuvre of the lower limbs to provide more information for the designs of athletic training protocol and footwear. Different lunging directions were studied, as they required different training attention as suggested by some other sports such as table tennis (Wong, Lee & Lam, 2020), running (Rasica et al., 2020), and gym (Apps et al., 2019). While footwear was proven a key effect modifier to the biomechanics of lunge (Apps et al., 2019; Wong, Lee & Lam, 2020), this review also studied the effects of footwear designs on the lunge.

The objective of this study was to exploit recent research methods and biomechanical data of badminton lunges and related performance. Since there are high complexity and heterogeneity across badminton lunge studies, it is impractical to report with a precise systematic review (Peters et al., 2015). Our systematic scoping review summarized a broad topic of evidence, examined how research is conducted on this topic, and categorized key concepts using a systematic approach (Munn et al., 2018). The research context and themes of this scoping review were mapped by the guided research questions as followed:

1. What were the methods and measures to analyze the biomechanics of badminton lunge?
2. What were the biomechanical attributes for higher-skilled players?
3. What were the biomechanical differences between manoeuvres and between footwear constructions?
4. How did the existing literature discuss the implications of injury from a biomechanical point of perspective?

This review categorized information into review context (research settings and protocols) and research themes (playing levels, the influence of footwear, and lunge directions/variations). The review context helped to inform the confounding factors, effect modifiers, and methodological disparities, while the research theme identified the knowledge gap and conflicting evidence (Munn et al., 2018; Peters et al., 2015) that require future studies to the badminton community. The information from this study can contribute to the field of sports science by attempting to extract key ideas and to inspire injury prevention, performance improvement, and footwear design.

## Survey Methodology

### Search strategy

Searches were designed and conducted by DWCW. Electronic literature searches of electronic databases included ISI Web of Science (excluding patents, from 1970), Scopus (from 1960), SPORTDiscus (from 1830), and PubMed (from 1975) was performed on 3 September 2020. The searches were made using a combination of the following keywords linked by the AND function: “badminton”, “lunge”, and “biomechan* OR kinematics OR kinetics” in the topic field of the databases. The title, abstract, and full-text of the included articles were screened based on the following inclusion criteria: (1) published in English; (2) in a peer-reviewed journal; (3) conducted experiments to investigate the badminton lunge manoeuvre of the lower limb; (4) biomechanical investigation in nature.

### Screening and study selection

The authors (DWCW and WKL) conducted screening on the abstract and full-text and subsequent data extraction. Any disagreements were resolved by discussion with the third author (WCCL), and all authors conducted a final check of the review.

An initial search identified 86 articles, and 54 duplicates were removed. Four articles were identified from grey literature. Fifteen articles were excluded because of non-original research articles, including review and survey only (*n* = 6); non-English article (*n* = 2); not related to lower limb biomechanics of lunge manoeuvre (*n* = 5); conducted on participants with pain or pathological conditions (*n* = 3). Finally, there were 20 articles eligible for inclusion after screening.

**Figure 1:**
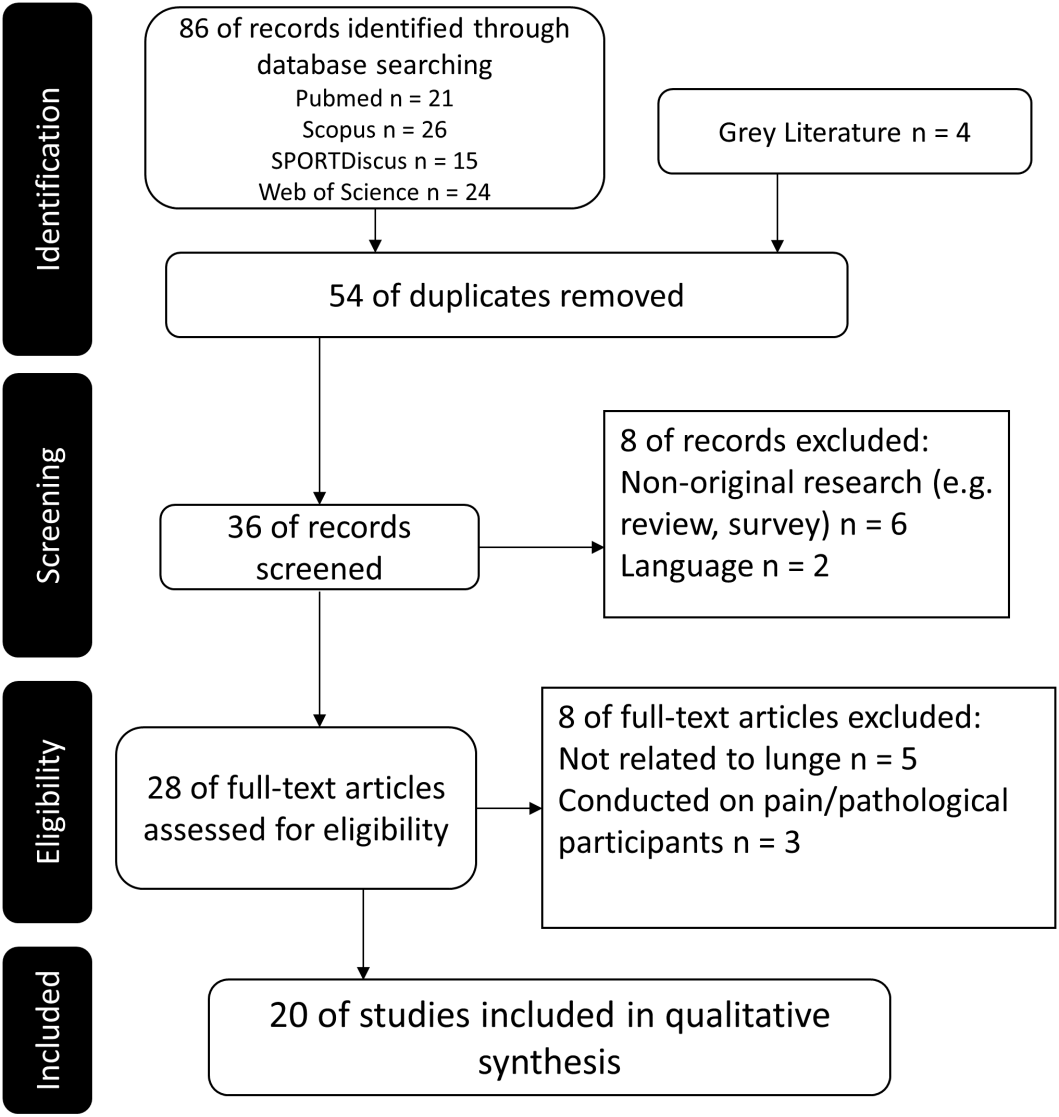
Flow chart of literature search and selection.

The search and study selection process is summarized in Fig. 1. Studies were excluded if they did not constitute any biomechanical outcome measures. There was no disagreement between authors in the selection of studies eligible for the review. The following data were extracted and grouped into the research context: bibliographic details, sample size, characteristics of participants, inclusion and exclusion criteria of studies, experimental settings, and outcome measures.

### Data extraction

Information of the 20 included studies qualified for further review is summarized based on the review context in Table 1 (population and selection criteria), Table 2 (experimental protocols), and Table 3 (outcome measures and key findings).

**Table 1:** Population and participant selection criteria in the included articles of badminton lunge.

Author (Year)	Sample size	Population Characteristics				Inclusion/exclusion criteria	
		Sex	Age/Height/Mass	Dominancy	Group/level		
Chen et al. (2020)	15	Male	25.8 yr (7.0) 171.4 cm (5.8) 66.3 kg (6.8)	Right-hand	Club level (>3 years exp.)	− Free from upper/lower limb injuries for at least 6 months	
Choi & Lee (2017)	15	Male	21.4 yr (3.0) 164.1 cm (7.9) 71.5 kg (6.8)	NS	University students	No nervous or cardiopulmonary system problems; No orthopaedic musculoskeletal issue related to trunk and lower limb.	
Fu, Ren & Baker (2017)	8	Male	23.4 yr (1.3) 172.7 cm (3.8) 66.3kg (3.9)	Right-hand, right-leg	Professionals	Members of the province club & participated at professional national lv.	Free from any injuries to both upper & lower limbs in the six-month period
	8	Male	22.5 yr (1.4) 173.2 cm (1.8) 67.5 kg (2.3)		Amateur	Completed for their college or university in inter-collegiate play	
Hong et al. (2014)	15	Male	21.69 yr (1.03) 172.61 cm (5.20) 61.67 kg (7.15)	Right-hand	<5 yrs of exp.	Shoe size EUR42; Active participants in single badminton competitions at the university lv.; Free from neuromuscular, vestibular & vision system injuries >6 months before participation, generally in good physical condition	
Hu et al. (2015)	15	Male	23.8 yr (3.3) 169.3cm (4.5) 62.67kg (8.1)	Right-hand	At least 2 yrs of competition exp.	Generally good physical condition; Actively participating in single badminton competitions at university lv.; Shoe size EUR 41; No visual problems, deformity in lower extremities or spine, previous history of surgery, neurological or systemic disorders; Did not take sedative drug or alcohol within the past 48 h	
Huang et al. (2019)	8	Male	23.4 yr (1.3) 172.7 cm (3.8) 66.3 kg (3.9)	Right-hand, right-leg	Professional 9.7 (1.2) yrs of exp.	Did not suffer from any injuries in the upper and lower limbs in the past six months; Did not take part in any high-intensity training or competition 2 day before the experiment.	
	8	Male	22.5 yr (1.4) 173.2 cm (1.8) 67.5 kg (2.3)		Amateur 3.2 (1.1) yrs of exp.		
Kuntze, Mansfield & Sellers (2010)	9	Male	20.0 yr (2.12) 179 cm (0.06) 70.58 kg (7.39)	NS	National lv.; <6 yrs of exp.	Actively taking part in single badminton competition	
Lam, Ding & Qu (2017)	13	Male	20.9 yr (1.0) 176 cm (0.05) 67.6 kg (5.3)	Right-hand	University players	Foot size US 9	Free from any lower extremity injury >6 months
Lam et al. (2017)	11	Male	20.6 yr (0.7) 176.0 cm (6.0) 70.9 kg (5.9)	Right-hand	Elite, 8.4 (1.4) yrs of exp.	Practicing with national team training	Free from any lower extremity injury >6 months
	15	Male	21.4 yr (1.6) 176.0 cm (6.0) 66.9 kg (5.7)		Intermediate, 3.2 (1.0)	Convenient participants from badminton club	
Lam et al. (2018)	11 11	Male Female	20.55 yr (0.68) 1.78 m (0.06) 70.91 kg (5.92) 21.91 yr (2.55) 1.67m (0.07) 60.82kg (5.74)	Right-hand	Skilled 8.36 (1.43) yrs of exp 10.09 (1.45) yrs of exp	Participated in international competitions, achieved Korean collegiate championship titles	Free from any lower extremity injuries >6 months
	15 15	Male Female	21.40 yr (1.55) 1.76 m (0.06) 66.93 kg (5.65) 21.60 yr (1.5) 1.64 m (0.04) 57.93 kg (5.98)		Unskilled 3.20 (1.01) yrs of exp 2.13 (0.64) yrs of exp	Average of 2-3 years badminton experience and less than 1-hour of playing per week; Did not take part in any formal competition	Free from any lower extremity injuries >6 months
Lund et al. (2017)	14	Male	24.8 yr (7.7) 178 cm (5) 72.6 kg (7)	NS	NS	NS	
Mei et al. (2014)	6	Male	NS	NS	Professional	NS	
Mei et al. (2017)	8	Male	23.4 yr (1.3) 172.7 cm (3.8) 66.3 kg (3.9)	Right-hand	Elite national-level 9.7 (1.2) yrs of exp.	Free from any injuries to the upper & lower limbs in the past half yr; Did not conduct any high-intensity training or competition 2 days before the experiment.	
	8	Male	22.5 yr (1.4) 173.2 cm (1.8) 67.5 kg (2.3)		Recreational, 3.2 (1.1) yrs of exp.		
Nadzalan et al. (2017)	15	Male	22.07 yr (1.39) 173.13 cm (2.12) 70.07 kg (1.88)	NS	University team players in badminton tournament	No medical problems	
Nagano et al. (2020)	10	Female	15.8 yr (1.0) 158.5 cm (3.4) 51.8 kg (3.9)	NS	NS	No history of serious musculoskeletal injury or any musculoskeletal injury within the past 3 months; Any disorder interfered with sensory input, musculoskeletal function, motor function.	
Nielsen et al. (2020)	14	Male	26.4 yr (5.5) 1.75 m (0.05) 69.8 kg (9.2)	Right-hand	5.5 (3.7) yrs of formal competition experience	Free of any lower extremity injuries in the past 6 months	
Park et al. (2017)	10	Male	19.7 yr (1.6) 176 cm (0.05) 70.4 kg (3.7)	NS	10.2 (1.8) yrs of exp.	Foot size US 9.0, >20 training hours/week; Free from any lower extremity injuries & foot deformities for the previous 6 months; did not have abnormal foot types or need foot orthotics	
Valldecabres et al. (2018)	10 6	Male Female	27.1 yr (9.0) 172.1 cm (8.9) 74.0 kg (16.5)	Right-hand	NS	Free from any pain or pathology affecting lower limbs	
Valldecabres et al. (2020)	5 8	Male Female	25.93 yr (10.05) Height: NS 64.30 kg (8.66)	Right-hand	First badminton league played at least 3 times a week	No injuries to upper/lower limbs in previous 6 months; No history of surgery or traumatic injury of the lower extremities or lower back; No history of medical conditions limiting physical activity; No neuromuscular, vestibular, visual impairment or back pain.	
Wei et al. (2009)	8	Male	19.9 yr (1.9) 177.5 cm (4.38) 72.25 kg (8.94)	NS	9.25 (3.92) yrs of exp.	Normal anatomical structures & function, adequate physical status & sports ability; lack of recent history of maximal training; No foot disease or injury	

**Notes.**

NS Not specific yr year lv. level exp. experience

**Table 2:** Experimental protocol for the biomechanical test for lunge maneuvers.

Author (Year)	Variant	Lunge direction		Shuttlecock placement/stroke	Footwear
Chen et al. (2020)	Lunge direction	Forward	Forehand Backhand	Hit the shuttlecock at the net	Li Ning (AYAE011)
Choi & Lee (2017)	Lunge types (4 different ankle abductions	Forward	Front	NS	NS
Fu, Ren & Baker (2017)	Playing lv.	Forward	Right	Underhand stroke	Same brand & series of badminton shoes
Hong et al. (2014)	Lunge direction vs. Two different brands of shoes	Forward Backward	Left Right Left Right	NS	NS (coded as Y & L shoe)
Hu et al. (2015)	Lunge direction vs. Three shoes with different brands	Forward	Front Left Right	Hit the shuttlecock [not mention stroke type]	Li Ning (2YMD649-1), Mizuno (7KM-75562), vs. Yonex (SHB-91MX),
Huang et al. (2019)	Playing lv.	Forward	Right	Hit the shuttlecock underhand to the backcourt	Same brand and series of shoes
Kuntze, Mansfield & Sellers (2010)	3 lunge types	Forward	Front	Threw the shuttlecock overnet & returned	NS
Lam, Ding & Qu (2017)	Single vs. repeated movement	Forward	Left	Shuttlecock suspended at 0.6m, drop shot	Li Ning SAGA
Lam et al. (2017)	Playing lv. vs. Three shoes with different heel curvatures	Forward	Left	Shuttlecock suspended at 0.6m	Rounded heel shoe, flattened heel shoe, standard heel shoe
Lam et al. (2018)	Playing lv. vs. gender	Forward	Right	Shuttlecock suspended at 0.6m	NS
Lund et al. (2017)	Insole hardness vs. region (rearfoot, midfoot, vs. forefoot	Forward	Right	Same experimental procedure as Hong et al. (2014) (NS)	Li Ning (AYAK23-2) with different insoles [soft (35C), medium (48C) & hard (60 C)]
Mei et al. (2014)	Two shoe sole stiffness	Forward	Right	NS	Sole stiffness 58 & 68 (Durometer Type C test)
Mei et al. (2017)	Playing lv.	Forward	Right	Underhand lift a threw shuttlecock	Same brand & series of badminton shoes
Nadzalan et al. (2017)	Lunge types (jump forward lunge vs. step forward lunge)	Forward	Front	NS	NS
Nagano et al. (2020)	Categorized 9 movements in game records (landing after an overhand stroke, lunging during an underhand stroke, cutting from a split step, take-off before overhand stroke, pre-split stepping, split step, stopping, back stepping, cutting)	NS	NS	NS	NS
Nielsen et al. (2020)	Lunge direction	Forward	Forehand Backhand	Shuttlecock was suspended 0.6m above the ground, at a distance of 0.60 m from the centre of force platform	NS
Park et al. (2017)	Same shoes with different forefoot bending stiffness (flexible, regular vs. stiff)	Forward	Right	Random assignment of either lunge & back-count high clear shot executed by a coach	Li-Ning, SAGA AYAZ005 original –market available, flexible shoe - a 2-mm thinner full-length mid-sole thickness, & stiff shoe - filled outsole flexing grooves at the forefoot.
Valldecabres et al. (2018)	Lunge direction vs. fatigue (pre- & post-) vs. brace (w/ & w/o)	Forward	Forehand Backhand	Shuttlecock positioned 0.15 in front of the net, 0.4 m to the side of the force plate at 1.65 m high; hit the shuttlecock with top spin shot	Own shoes; off-the-shelf proprioceptive knee brace for the brace condition
Valldecabres et al. (2020)	Lunge direction vs fatigue (pre- & post-)	Forward	Dominant & Non-dominant directions	Hit the shuttlecock with a top-spin shot	NS
Wei et al. (2009)	Barefoot vs. other two shoe types	Forward	Right	Catch the shuttlecock	SHB-99 male Yonex, New shoe design for Asians

**Notes.**

NS Not specific vs. versus w/ with w/o without

**Table 3:** Outcome measures & key findings of the reviewed articles.

Author (Year)	Endpoints	Outcome parameters	Findings
Chen et al. (2020)	Kinetics	Peak vGRF & hGRF, peak contact force of hip, knee & ankle	Compared to forehand forward lunge, backhand forward lunge: ↓ ankle joint force in compression direction; ↓ Peak deceleration of total mass centre & torso in horizontal direction during braking; ↓ hip abduction angular velocity at touchdown; ↑ hip frontal plane RoM during lunge stance.
	Kinematics	Velocity at touchdown, peak deceleration during braking & displacement during lunge stance for total body mass centre, torso, pelvis, thigh, shank & foot, angular velocity at touchdown, peak angular deceleration during braking & RoM during lunge stance for lower-lumbar, pelvis, hip, knee & ankle.	
Choi & Lee (2017)	Muscle activity	EMG of Rectus femoris (RF), vastus lateralis (VL), vastus medialis oblique (VMO)	Increased ankle abduction in lunge ↑ muscle activity of RF, VL & VMO.
Fu, Ren & Baker (2017)	Kinematics	RoM of knee & ankle in all planes	Amateurs ↑ ankle ROM, inversion & internal rotation joint moment. Professionals ↑ knee joint moment in the sagittal & frontal plane. Professionals ↑ vGRF at the drive-off phase.
	Kinetics	vGRF; knee & ankle moment in all planes; during 1st & 2nd impact peak, weight acceptance & drive-off phases	
Hong et al. (2014)	Kinetics	1st & 2nd peak of vGRF, max AP shear, time to peak GRF, max LR.	↔ shoe effects. Left-forward lunge had: ↑ 1st vGRF than that of right-backward & left-backward lunge; ↑2nd vGRF than that of left-backward lunge; ↑max AP shear force than that of left-backward lunge; highest mean vGRF & AP shear, & respective max loading rates; highest mean peak pressure at the total foot & heel regions; Right-backward lunge had highest peak pressure at the midfoot & medial MT heads.
	Plantar pressure	Peak pressure at total foot, medial heel, lateral heel, medial midfoot, lateral midfoot, 1st MT head, 2nd to 3rd MT heads, 4th to 5th MT heads, great toe, lesser toes.	
Hu et al. (2015)	Plantar pressure	Peak pressure, maximum force & contact area of medial heel, lateral heel, medial midfoot, lateral midfoot, medial forefoot, central forefoot, lateral forefoot, great toe, lesser toes.	↔shoe effects. Front-forward lunge ↓ maximum force & peak pressure on the great toe than left- & right-forward lunge. Left-forward lunge ↓maximum force on the lateral midfoot than front-forward lunge.
Huang et al. (2019)	Kinematics	Knee & ankle RoM in all planes during initial impact, secondary impact, weight acceptance & drive-off phases	Professional players showed: ↑ vGRF during drive-off; ↓ ankle RoM in the frontal plane; ↑ ankle & knee RoM in the transverse plane; ↑ mean ankle moment in the sagittal plane during secondary impact; ↑ mean ankle moment in the frontal plane during weight acceptance; ↑ mean knee moment in the frontal plane during initial impact; ↓ mean knee moment in the transverse plane during drive-off.
	Kinetics	Peak vGRF, Mean knee & ankle moment all planes during initial impact, secondary impact, weight acceptance & drive-off phases	
Kuntze, Mansfield & Sellers (2010)	Spatiotemporal parameters	Approach speed, total duration, stance phase duration, recovery duration	Hop lunge: ↑stance phase duration than other lunge conditions; ↓recovery duration than step-in lunge; ↑peak vGRF & hGRF among the lunge conditions; ↑2nd peak ankle plantarflexion moment among the lunge conditions.
	Kinematics	Peak hip, knee & ankle sagittal angles	
	Kinetics	vGRF, hGRF during 1st & 2nd impact peaks, amortisation, weight acceptance & drive-off phases; Hip, knee & ankle sagittal moment	
Lam, Ding & Qu (2017)	Spatiotemporal parameters	Mean & CV of approaching speed, contact time	Repeated lunge: ↑ peak hGRF; ↓ contact time, max vGRF LR; smaller knee AP force & peak knee sagittal moment; ↓ CV for peak knee ML & vertical forces.
	Kinematics	initial shoe-ground angle	
	Kinetics	Peak vGRF & hGRF	
Lam et al. (2017)	Spatiotemporal parameters	Approaching time, contact time, CV of the above parameters	↔interaction effect on spatiotemporal parameters, knee moments, & CVs between the playing lv.s & shoe design conditions. Significant interaction between playing lv.s & shoe design on the max & mean loading rates of vGRF. Rounded heel shoe had the lowest max vGRF LR, followed by the standard heel shoe, & then the flattened heel shoe. Elite players showed ↑sagittal footstrike angle, peak knee flexion & extension moments; ↓CV for contact time, sagittal footstrike angle, peak vertical impact force, mean LR, but ↑ CV for max resultant transverse LR than intermediate group.
	Kinematics	Sagittal footstrike angle, CV of sagittal footstrike angle	
	Kinetics	Peak vGRF & hGRF, max & mean vertical LR, max &resultant transverse LR; Peak knee extension & flexion moments, CV of above parameters	
	Kinetics	Mean & CV of Peak vGRF & hGRF, mean & max vGRF & hGRF LR in braking phase, Knee sagittal, frontal & transverse forces & moments	
Lam et al. (2018)	Spatiotemporal parameters	Approaching speed, foot contact time, maximum lunging distance	↔interaction effect on spatiotemporal parameters, footstrike angle, peak hGRF, loading rates & peak knee flexion moment. Significant interaction between gender & playing lv. on peak vGRF & peak knee extension moment. Gender effect showed: −↑ approaching speed, max lunge distance, max & mean loading rates, peak knee flexion moment for male. Skilled players showed: ↓ foot contact time, peak hGRF; ↑ footstrike angle, max loading rate
	Kinematics	Footstrike angle	
	Kinetics	Peak vGRF & hGRF, max loading rate, mean loading rate; Peak knee extension & flexion moments	
Lund et al. (2017)	Muscle activity	Estimated positive work for the passive, tendon & muscle elements of the gastrocnemius	↔insoles conditions for positive work of passive, tendon & muscle elements.
Mei et al. (2014)	Spatiotemporal parameters	Time to peak vGRF, heel landing time	↔vGRF & landing time between hardness conditions. Soft sole condition ↑ time to peak VGRF than hard sole condition.
	Kinetics	vGRF	
Mei et al. (2017)	Kinematics	Peak ankle & knee angles in all planes	Professional players: ↑ peak pressure on medial forefoot & hallux, & ↑ PTI on the medial forefoot; ↓ peak pressure on the lateral rearfoot & lateral forefoot, & ↓ PTI on the lateral rearfoot, lateral forefoot & other toes; ↑ peak ankle eversion & rotation, but ↓ peak ankle plantarflexion; ↑ peak knee internal rotation.
	Plantar pressure	Peak pressure & pressure time-integral (PTI) in medial rearfoot, lateral rearfoot, medial midfoot, lateral midfoot, medial forefoot, lateral forefoot, hallux & other toes	
Nadzalan et al. (2017)	Kinetics	GRF: Absolute peak concentric force, relative peak concentric force, absolute mean concentric force, relative mean concentric force, absolute mean eccentric force, relative mean eccentric force, absolute impact force, relative impact force, time to peak force, stance time	Jump forward lunge, for both dominant & non-dominant limb −↑ all kinetic variables, compared to that of step forward lunge. Dominant limb, for both step forward & jump forward lunge; ↑ all force variables, compared to that of the non-dominant limb; ↓ all time variables, compared to that of the non-dominant lim.
Nielsen et al. (2020)	Kinetics	vGRF during initial impact peak, secondary impact peak, amortisation & weight acceptance; total impulse during foot contact; hip, knee & ankle moments in all planes	Forehand lunge had: ↑ ankle, hip & knee moment in the frontal plane; ↓ total impuse during contact, hip & knee moment in the transverse plane.
Nagano et al. (2020)	Kinematics	Resultant, vertical, ML, AP acceleration	Compared to other movements, lunging during an underhand stroke with the dominant-hand side leg: ↓ resultant acceleration than landing after an overhand stroke on the dominant-hand side leg; ↑ ML acceleration than all other movements; ↓ vertical acceleration than all other movements; ↑ AP acceleration than all other movements except cutting from a split step using the dominant-side leg.
Park et al. (2017)	Kinematics	Peak shoe torsion, shoe bending, ankle sagittal, frontal & transverse angles; RoM of shoe torsion, shoe bending, RoM of ankle in all planes	↔in all tested biomechanical variables, but the flexible forefoot outsole ↓ perception of comfort in forefoot cushion than regular & stiffer forefoot outsoles.
Valldecabres et al. (2018)	Spatiotemporal parameters	Approach velocity, right stance time	↔ interaction effect between factors for any variables Significant main effect b/n pre- & post- fatigue −↓ knee flexion angular velocity at heel strike & range of knee angular velocity in the coronal plane, stance time, knee abduction moment, range of moment in the coronal plane during post-fatigue. Significant main effect b/n brace & no brace −↓ peak knee adduction moment with brace condition. Significant main effect b/n backhand & forehand; ↑ knee flexion; ↓ internal rotation velocity; ↓ knee extension moment during forehand lunge.
	Kinematics	Peak knee joint angle & angular velocity in flexion, valgus, varus, external & internal rotation & at heel strike instant. Knee RoM & range of angular velocity in all planes.	
	Kinetics	Peak vertical force, loading rate; Peak knee joint moments in flexion, extension, adduction, abduction, internal & external rotation. Moment range in all planes.	
Valldecabres et al. (2020)	Plantar pressure	Peak & mean pressure for the lead & trail feet under hallux, 2nd–3rd phalanges, 4th–5th phalanges, 1st MT, 2nd–3rd MT, 4th -5th MT, medial midfoot, lateral midfoot & calcaneus regions.	Significant interaction effect between lunge directions & fatigue states in peak pressure of 2nd -3rd MT region of lead foot & 2nd -3rd phalanges region of trail foot. Significant main effect fatigue state in: ↓ peak & mean pressure under 4th -5th phalanges region for lead foot after fatigue; ↓ peak & mean pressure under 1st MT region for trail foot after fatigue; ↓ mean pressure under hallux region for trail foot after fatigue; ↑ peak & mean pressure under medial midfoot for trail foot after fatigue. Significant main effect lunge direction in: ↓ mean pressure under calcaneus in dominant-side lunge for lead foot; ↑ peak & mean pressure under hallux & 2nd-3rd phalanges region in dominant-side lunge for trail foot.
Wei et al. (2009)	Spatiotemporal parameters	Time of max MTP in pedal & stretch phases	Barefoot condition ↑ MTP angle at heel lift, static angle of MTP in propulsion phase, MTP angle at moment of pedal & stretch phases, & longer time of max MTP in pedal & stretch phases, compared to the Yonex & prototype shoes. Yonex ↓ MTP angle at heel lift. & prototype shoes only showed significant difference on MTP angle at heel lift.
	Kinematics	MTP angle &angular velocity at heel lift, MTP angle & max MTP angular velocity in pedal & stretch phases, MTP angle at moment & end of pedal & stretch phases, static angle of MTP in propulsion.	
	Kinetics	MTP joint stiffness in the pedal & stretch phases	

**Notes.**

↔ no significant ↑ significantly larger ↓ significantly smaller AP anteroposterior CV coefficient of variation EMG Electromyograph GRF ground reaction force HSA hip-shoulder separation angle hVGF resultant horizontal ground reaction force LR loading rate MT metatarsal MTP metatarsophalangeal MVIC maximum voluntary isometric contraction RMS Root-mean-square RoM Range of Motion SAA shoulder-arm separation angle TTA trunk tilting angle vGRF vertical ground reaction force lv. level b/n between

### Methodological quality assessment

The methodological quality of each study was assessed using the modified Downs and Black Quality Index Tool (Downs & Black, 1998). The scale consists of 27 questions evaluating the quality of reporting, external and internal validity, and statistical power. It was proven to demonstrate high internal consistency (Downs & Black, 1998). In this study, seven questions (items 8, 9, 13, 17, 19, 25, 26) were not applicable due to the study design of this review (Richmond et al., 2013). Additionally, the power subscale (item 27) was discarded due to ambiguity, as recommended in the literature (Deeks et al., 2003). Therefore, a 19-item scale was used to evaluate the study quality.

The quality test was conducted by the authors (DWCW & WKL) independently. Any disagreements found were resolved by discussion with the third author (WCCL). The authors agreed to refine and remark some items, as stated in Table 4, to avoid ambiguity. The findings of the methodological quality assessment were addressed in the discussion section.

**Table 4:** Assessment of methodological quality of reviewed studies using the Modified Downs and Black Quality Index Tool.

**Item/ Study**	Chen et al. (2020)	Choi & Lee (2017)	Fu, Ren & Baker (2017)	Hong et al. (2014)	Hu et al. (2015)	Huang et al. (2019)	Kuntze, Mansfield & Sellers (2010)	Lam, Ding & Qu (2017)	Lam et al. (2017)	Lam et al. (2018)	Lund et al. (2017)	Mei et al. (2014)	Mei et al. (2017)	Nadzalan et al. (2017)	Nagano et al. (2020)	Nielsen et al. (2018)	Park et al. (2017)	Valldecabres et al. (2018)	Valldecabres et al. (2020)	Wei et al. (2009)
**1**	1	1	1	1	1	1	1	1	1	1	1	0	1	1	1	1	1	1	1	1
**2**	1	1	1	1	1	1	1	1	1	1	1	1	1	1	1	1	1	1	1	1
**3**	1	0	1	1	1	1	0	1	1	1	0	0	1	0	0	1	0	1	1	0
**4**	1	0	1	0	0	1	1	1	1	1	0	0	1	0	1	1	1	1	1	0
**5**	1	0	1	1	1	0	1	1	0	1	0	0	1	0	1	1	1	1	1	0
**6**	1	1	1	1	1	1	1	1	1	1	1	0	1	1	1	1	1	1	1	1
**7**	1	1	1	1	1	0	0	1	1	1	0	0	0	1	1	1	1	1	1	1
**10**	1	0	1	0	0	0	1	1	1	1	1	1	1	0	0	1	1	1	1	1
**11**	1	0	0	0	0	0	0	0	0	0	0	0	0	0	0	0	0	0	1	1
**12**	1	0	1	1	1	1	1	1	0	1	0	0	1	1	0	1	1	0	1	1
**14**	0	0	0	0	1	0	0	0	0	0	0	0	0	0	0	0	0	0	0	0
**15**	0	0	0	0	0	0	0	0	0	0	0	0	0	0	0	0	0	0	0	0
**16**	1	1	1	1	1	1	1	1	1	1	1	1	1	0	1	1	1	1	1	1
**18**	1	1	1	1	1	1	1	1	1	1	1	1	1	1	0	1	1	1	1	1
**20**	1	1	1	1	1	1	1	1	1	1	1	1	1	1	1	1	1	1	1	1
**21**	0	1	1	0	0	0	1	1	0	0	0	0	0	1	0	0	0	0	1	1
**22**	0	0	0	0	0	0	0	0	0	0	0	0	0	0	0	0	0	0	0	0
**23**	1	0	0	0	1	0	1	1	1	0	1	0	0	1	0	1	1	1	0	0
**24**	0	0	0	0	0	0	0	0	0	0	0	0	0	0	0	0	0	0	0	0
**Total**	14	8	13	10	12	9	12	14	11	12	8	5	11	9	8	13	12	12	14	11

**Notes.**

1 Yes 0 No

Item 1: hypothesis, aim, objectives; Item 2: main outcome; Item 3: subject characteristics (the characteristics of patients were clearly described only if dominancy was stated); Item 4: intervention (the interventions were clearly described only if the procedure/protocol was clearly described); Item 5: confounders (the principal confounders were clearly described if it was addressed in the discussion. The scoring method was modified as yes = 1 and no = 0); Item 6: main findings; Item 7: random variability estimates (error bars in graphs were not regarded as clearly providing the estimates of variability); Item 10: probability values; Item 11: Source population of the sample and clear selection criteria; Item 12: representative of the population, (the samples were regarded as representative if their levels of playing were clearly stated); Item 14: blinded from subjects; Item 15: blinded from experimenter; Item 16: data dredging; Item 18: appropriate statistical test; Item 20: accurate outcome measures; Item 21: recruitment from same population; Item 22: recruitment from same time frame; Item 23: randomization; Item 24: double-blind intervention assignment.

## Review Context

### Participant characteristics

There were a total of 314 participants (264 males, 50 females) in the 20 included articles (Table 1). Sixteen of them included male participants only. One article investigated only female participants. Only one article had participants with male-to-female at a one-to-one ratio (Lam et al., 2018). The sample size of each subject group ranged from 4 to 17. All participants were young healthy adults with an average age ranged from 19.9 to 27.1 years old. Participants were generally excluded from musculoskeletal problems, previous or current injuries, or surgeries. Moreover, some included articles required the participants to have an uncorrected vision and free of medications and alcohol. The skill levels of participants were classified based on the years of experience and levels of badminton competitions.

### Experimental protocol

Lunge manoeuvres can be performed in five directions, including three forward directions (forehand, backhand, and in-front) and two backward directions (forehand and backhand). Almost all included articles examined lunge biomechanics in forward direction, while one did not specify the direction of lunge (Nagano et al., 2020). Three included articles considered backward lunges, and six compared the movement mechanics among lunge directions. Hong et al. (2014) examined the differences in the impact of vertical and horizontal ground reaction forces (GRF) among lunges in four diagonal directions, while some included articles compared forehand and backhand performance in forward direction (Chen et al., 2020; Nielsen et al., 2020; Valldecabres et al., 2018). Moreover, Hu et al. (2015) compared the plantar loading pattern amongst three forward lunges (forehand, backhand, and in-front).

### Variation in lunge manoeuvre

The kick lunge, consisting of three steps, was commonly investigated in badminton studies on lower limb biomechanics (Table 2). The first step was to start from the initial position in standing or before two running steps. The next step was to launch the lunging step striking on the force plate and hit the target (shuttlecock). Two included articles required the participants to explicitly extend the knee (i.e., lunging leg) in the second step to achieve the maximum lunge distance (Lam, Ding & Qu, 2017; Lam et al., 2017), while one included article evaluated the sensitivity of ankle abduction angle (Choi & Lee, 2017). The third step (recovery phase) was to return to the initial position as fast as they could in gameplay. Before the experiment, the coaches (or elite athletes) who were able to consistently reproduce the manoeuvre demonstrated the footwork and placement to less skilled participants. In addition, the badminton coach and the experimenters confirmed whether a trial was successive and valid (Hong et al., 2014; Hu et al., 2015).

Furthermore, there were some other testing requirements when performing the lunge manoeuvre. Lam et al. (2018) controlled the total completion time of the move (forward lunge + recovery) within 3 s. The lunge distance between the starting position and force plate was determined as 2.5 times the foot length during a forehand forward lunge and three times of the foot length during a forehand backward lunge, respectively (Hu et al., 2015).

Three included articles investigated different variations of lunges. Nadzalan et al. (2017) asked the participants to perform both step-lunge and jump-lunge, which were achieved by bending the trunk anteriorly at 45° with thigh maintained horizontal, and by jumping explosively during the lunge and return, respectively. In addition, Kuntze, Mansfield & Sellers (2010) compared the step-in and hop lunges to the kick lunge. Step-in lunge was characterized by pulling the non-dominant limb towards the dominant limb at recovery to raise the body, while the hop lunge incorporated a hop after the strike and before the recovery. Lam, Ding & Qu (2017) highlighted that athletes were always moving back and forth during a game, and considering a single isolated lunge may not be realistic, despite that there were challenges to define and segment phases and events of movement accurately. Nagano et al. (2020) considered the more realistic aspect of the game. They extracted and classified the frequency of different movements in the game that included landing after an overhand stroke, lunging during an underhand stroke, cutting from a split step, take-off before the overhand stroke, pre-split steeping, split step, stopping, backstepping, and cutting. One of the drawbacks was that the movement could not be controlled, and the directions of the lunge were not specified to allow comparison across studies.

### Shuttlecock position/task

The assignments of the hitting target (shuttlecock) were also different among studies, which might alter both upper and lower limb biomechanics of a lunge. During the experiments, a shuttlecock was often suspended at 0.6 m height as the hitting target (Lam, Ding & Qu, 2017; Lam et al., 2018; Lam et al., 2017; Nielsen et al., 2020; Valldecabres et al., 2018). On the other hand, Kuntze, Mansfield & Sellers (2010) and Mei et al. (2017) manually threw the shuttlecock over the net to a dedicated court area. Five included studies did not mention the use of shuttlecock nor address how it was positioned. Athletes were asked to perform an underhand stroke to resemble a frontcourt shuttle-drop or drop-shot serve conditions. More precisely, Lam, Ding & Qu (2017) instructed the participants to grip the racket backhand or thumb-up in front of the body and executed a drop-shot by lifting motion that only allowed a small amount of shoulder follow-through (Grice, 2008).

### Footwear construction

The influence of footwear construction is undeniable since it is associated with the plantar loading pattern and landing kinematics during lunges. Some included articles did not report if footwear was standardized. Valldecabres et al. (2018) reported that their participants wore their own badminton shoes, whereas Fu, Ren & Baker (2017), Mei et al. (2017), and Huang et al. (2019) provided the same brand and series of badminton shoes used in the experiments. Several included articles utilized the badminton shoes from the Li Ning (Hu et al., 2015; Lam, Ding & Qu, 2017; Lund et al., 2017; Park et al., 2017), Mizuno, and Yonex company (Hu et al., 2015; Wei et al., 2009). Only one included article attempted to compare between barefoot and shod conditions that introduced a prototype designed for the foot shape of Asian players (Wei et al., 2009).

Isolated footwear design/constructions were also investigated in heel curvature design (Lam et al., 2017), midsole thickness (Lin et al., 2016), sole hardness (Mei et al., 2014), insole hardness (Lund et al., 2017), and forefoot bending stiffness (Park et al., 2017). Lam et al. (2017) examined the effect of the geometry of shoe heel curvatures (rounded, standard, and flattened heel) during lunge in elite and intermediate athletes. Lin et al. (2016) hypothesized that thicker midsoles might decrease joint instability and performance and thus tested this contention with shoes with three different midsole thickness. Moreover, Lund et al. (2017) divided the insoles into three plantar regions (rearfoot, midfoot, and forefoot) to further investigate the effect of the forefoot and rearfoot hardness on joint mechanics during lunges. While keeping the midfoot region as medium hardness, Lund et al. (2017) evaluated the insoles with five combinations of regional hardness with three hardness levels (hard, medium, and soft). Regarding midsole hardness, Mei et al. (2014) compared two hardness of soles (58 vs 68) using a Durometer (Type C) on lower limb biomechanics. On the other hand, Park et al. (2017) reduced the forefoot bending stiffness of a shoe by reducing the midsole thickness by 2 mm, while increased the stiffness by filling the outsole flexing grooves. In summary, some reviewed articles did not provide details on the selection of footwear in the experiment, while some attempted to use commercially available badminton shoes. For articles comparing footwear construction factors, midsole thickness, midsole hardness, and heel curvature were of concern in badminton lunge.

### Outcome measures

#### Spatiotemporal parameters

Agility was often assessed by approach speed (or time), foot contact time, and total completion time during lunges since participants were instructed to return to the initial starting position as fast as possible (Kuntze, Mansfield & Sellers, 2010; Lam, Ding & Qu, 2017; Lam et al., 2018; Lam et al., 2017; Lin et al., 2016; Valldecabres et al., 2018). The approach speed was the average speed lunging from the starting position to the initial contact of the lunging leg (i.e., racket side) (Lam et al., 2018). Afterward, the contact time (or stance duration) was calculated from the initial lunge landing until the foot take-off for recovery. The recovery duration was proposed by Kuntze, Mansfield & Sellers (2010) to quantify how fast the athletes could return. Time to peak GRF and loading rates were also used to quantify the degree of the landing impact (Lin et al., 2016; Mei et al., 2014; Nadzalan et al., 2017).

#### Kinematics

Range of motion (RoM) was defined as the difference between maximum and minimum angles of the hip, knee, and ankle joints, which were commonly evaluated at lunge, hitting, and recovery phases. Fu, Ren & Baker (2017) measured knee and ankle RoM to identify the differences between amateur and professional athletes. Park et al. (2017) investigated peak and changes in shoe bending and torsion angles between the forefoot and rearfoot regions, together with ankle RoM, to examine how forefoot bending stiffness of footwear would influence foot and ankle mechanics. Moreover, Nagano et al. (2020) extracted and classified badminton movements when a resultant acceleration of more than four times of gravity was generated under the premise, resulting in injuries to trunk or body segments. In addition, Chen et al. (2020) measured segmental and angular segmental deceleration as a sign of better performance. A high-level of deceleration enabled quick recovery from lunges and was facilitated by high eccentric muscle forces and core stability.

Landing angle (or footstrike angle) can represent the mechanism and characteristics of landing, particularly influenced by footwear, gender, and playing level (Hong et al., 2014; Lam, Ding & Qu, 2017; Lam et al., 2018; Lam et al., 2017). The ankle, knee, and hip kinematics were also taken into account for the understanding of lunge characteristics. Lin et al. (2016) attempted to examine the coordination between midfoot pronation and ankle inversion. On the other hand, Wei et al. (2009) stressed the importance of the pedal-and-stretch process as one of the major fundamental sports movements. It was defined as the active stretching of the joints that generate the push-off force on the ground and hence promote foot and whole-body movement. Resembling a pedal-and-stretch process of the foot and ankle, Wei et al. (2009) appraised the angle and angular velocity of the metatarsophalangeal joint. The forefoot stiffness of the foot was also quantified by the ratio of maximum GRF relative to the angular deflection of the metatarsophalangeal joint (Oleson, Adler & Goldsmith, 2005; Wei et al., 2009).

#### Kinetics

The mean and peak vertical and horizontal GRF, the loading rates were of interest (Table 3). The loading rate of GRF was defined as the slope of the force, which was determined from 20% to 90% of the impact force peak magnitude (Lam, Ding & Qu, 2017). Lin et al. (2016) subdivided lunge movement into braking and propulsion impulses, in which the cut-off was divided at the valley point (lowest value after the first peak) of the vertical GRF. Hong et al. (2014) discovered two peaks in the vertical GRF curve, in contrast with the findings of Lin et al. (2016), which found three vertical peaks in their GRF curves. A possible explanation could be the differences between amateur and professional athletes, as indicated by a significant difference in the vertical GRF during the late stance of the lunge (Fu, Ren & Baker, 2017). Different playing levels showed significant differences in peak vertical and horizontal GRF, whereas gender would play a role in maximum and mean loading rates of the impact peak (Lam et al., 2018).

Kuntze, Mansfield & Sellers (2010), Huang et al. (2019), and Nielsen et al. (2020) suggested that different variations of lunge manoeuvres also led to different GRF patterns. GRF can be divided into the initial impact peak, secondary impact peak, amortization, weight acceptance, and drive-off phases. Complementary, joint moment and power of the lower limb were also determined by the GRFs and joint position and mechanics (Fu, Ren & Baker, 2017; Kuntze, Mansfield & Sellers, 2010; Lam, Ding & Qu, 2017; Lam et al., 2017). Despite Chen et al. (2020) believed that joint moments might not directly reflect joint loading and endeavored to estimate joint contact forces using musculoskeletal computational models. The understanding of kinetic parameters could provide better insights on biomechanical requirements and the contribution of different joints during a motor task (Kuntze, Mansfield & Sellers, 2010). Analyzing joint moment and power variables could better indicate muscle strength, control strategy, stability weight transfer capability that distinguished higher-level players (Fu, Ren & Baker, 2017; Lam, Ding & Qu, 2017). Elite athletes managed to utilize higher vertical GRF, instead of the loading rate of vertical GRF, to achieve better performance and prevent injury (Zadpoor & Nikooyan, 2011).

#### Plantar pressure

Plantar pressure was investigated in four included articles (Hong et al., 2014; Hu et al., 2015; Mei et al., 2017; Valldecabres, Richards & De Benito, 2020). Typically, the footprint was divided into lateral and medial rearfoot, lateral and medial midfoot, metatarsal, hallux, and other toes regions to study plantar loading between interventions. Regarding the forefoot region, the region can be sub-divided into medial and lateral sides (Mei et al., 2017) or medial, central, and lateral regions (Hong et al., 2014; Hu et al., 2015). Peak pressure, maximum force, pressure–time-integral, and contact area were common parameters to evaluate plantar pressure profiles in the included articles. These outcome parameters describe the quality and efficiency of footwork in addition to the loading strategy and fatigue conditions (Valldecabres, Richards & De Benito, 2020). For example, the speed on reaching the shuttlecock, impact attenuation capability, and movement stabilization to avoid injury (Hu et al., 2015).

#### Muscle activity

Muscle activity of lower extremity was evaluated using either electromyography (EMG) or musculoskeletal model. Choi & Lee (2017) evaluated the EMG of the rectus femoris, vastus lateralis, and vastus medialis oblique in different angles of ankle abduction during lunges. Using a computational simulation approach, Lund et al. (2017) input the kinematic and kinetic data into a musculoskeletal model to determine the positive work of passive tendon and muscle elements for gastrocnemius and soleus. There were relatively fewer studies done on muscle activity.

## Research Themes

### Differences in playing levels

Five included articles investigated biomechanical differences between playing levels. While both amateur and professional badminton athletes showed similar maximum lunge distances, professional athletes demonstrated larger footstrike angles and faster approaching speeds that could characterize as better performance (Lam et al., 2017). Better heel impact attenuation during the landing of a lunge step was another attribute of professional athletes, which is demonstrated by smaller peak horizontal GRF and loading rates, although comparable vertical GRF was noted compared to the amateurs (Lam et al., 2017). Another included article from the same research group indicated that professional athletes produced significantly smaller peak vertical and horizontal GRF, whilst loading rates were mainly governed by the gender factor (Lam et al., 2018). Professional athletes landed at the lateral heel of the lunging foot and gradually shifted plantar load to the medial forefoot and hallux regions from landing to the drive-off phase (Kuntze, Mansfield & Sellers, 2010). They showed significantly higher peak pressures in the medial forefoot and hallux regions, and higher force-time integrals in the medial forefoot. This was compared to amateur athletes who presented significantly higher peak pressures and force-time integrals in the lateral forefoot and lesser toe regions (Mei et al., 2017). Abnormal high loading at the lateral side of the foot might indicate an inverted ankle landing posture, which could be a potential contributor for ankle inversion sprain and associated ligament injuries (Shariff, George & Ramlan, 2009).

Professional athletes generally elicited greater mechanical outputs at the knee and ankle that aided to transfer body mass effectively (Fu, Ren & Baker, 2017). The professional players demonstrated greater knee joint moments in both sagittal and frontal planes (Fu, Ren & Baker, 2017; Huang et al., 2019; Lam et al., 2017), greater peak knee adduction, and internal rotation angles (Mei et al., 2017). To allow quick recovery from lunges, athletes exhibited smaller knee and hip flexion angles, which were associated with shorter stance times (Brahms, 2014; Mei et al., 2017). On the other hand, interestingly, opposite findings indicated that professional athletes demonstrated both larger (Lam et al., 2017) and smaller (Lam et al., 2018) peak knee flexion moment and impact loading rate. The former was explained by greater power production while the latter was explained by better efficiency in elite athletes. Similarly, there are conflicting results related to ankle joint variables across included studies. Fu, Ren & Baker (2017) found that amateurs produced greater ankle inversion and internal rotation angles, whereas Mei et al. (2017) found amateurs had smaller peak ankle inversion and ankle internal rotation, but larger peak plantarflexion angle and RoM. Huang et al. (2019) found that professional athletes produced greater ankle eversion moment during weight acceptance phase. Poorer muscle control yielding ankle instability may lead to a larger range of motion (Fu, Ren & Baker, 2017). In contrast, Mei et al. (2017) and Huang et al. (2019) attributed the findings due to poor landing techniques and fatigue of their participants. Valldecabres et al. (2018) showed that fatigue caused a significant reduction in knee angular velocity during heelstrike, range of knee angular velocity in the coronal plane, and knee abduction moment. Discrepancies could be due to the differences in specific tasks, difficulty, or instructions. For example, the direction of lunges, repeated or isolated lunges (Lam et al., 2018; Lam et al., 2017), instructions on whether the lunge would perform maximally or replicate according to a model participant (Fu, Ren & Baker, 2017; Mei et al., 2017). Further investigation is warranted, and the interaction with footwear could be a plausible explanation (Lam et al., 2017).

### Influence of footwear

Our included articles did not demonstrate any promising evidence of the influence of midsole hardness or forefoot bending stiffness on lunge biomechanics. A softer midsole is suggested to provide better cushioning, lower peak vertical GRF, and longer landing time, but some included articles did not show significant differences among various midsole hardness conditions (Mei et al., 2014). Similar findings were obtained from a computational model investigating different insole hardness (Lund et al., 2017). Moreover, different shoe bending stiffness did not impose observable alteration on the ankle and foot kinematics, even though perceived cushioning can be altered (Park et al., 2017). It could be explained by “comfort filter” and “preferred movement path” paradigms, which suggested that the human body could unconsciously select comfortable footwear based on their own perception to allow for individual preferred movement path (Nigg et al., 2015). Athletes could optimize muscle forces to complete the lunge movement successfully and to reduce excessive impact forces (Lees & Hurley, 1994).

Compared to the barefoot condition, wearing sports shoes reduced metatarsophalangeal joint flexion and elongated length of plantar muscles that may enhance push-off efficiency (Wei et al., 2009). However, different badminton shoe models used did not significantly change the footstrike angles, GRF, and plantar load distribution (Hong et al., 2014; Hu et al., 2015). On the other hand, Lin et al. (2016) found the shoes with thicker midsoles were unable to influence shock attenuation, but in another study, thicker midsoles were found to be associated with impaired proprioception (Robbins et al., 1994), leading to higher peak midfoot pronation at the early stance that may be related to overuse injuries. In a similar vein, heel geometry design was identified to be one plausible construction to improve impact attenuation capacity during lunge (Lam et al., 2017). Rounded heel shoes could reduce maximum vertical loading rates compared to flat or standard heels shoes. However, such a situation was only applicable to elite athletes but not to amateurs, which may imply that professional athletes were more sensitive and adapted to very small changes in footwear. In summary, softer or thicker footwear may not be effective in shock absorption despite providing better comfort perception. The impact of forefoot bending stiffness and heel curvature on lower limb biomechanics may be influenced by some confounding factors, such as the level and accommodation capability of the athletes. Future studies should investigate different material and structural designs that could compromise impact attenuation, proprioception, and performance.

### Lunge directions and variations

The left (backhand/non-racket side) forward lunge is suggested to be the most critical as this lunge direction had the highest plantar loading than other lunge directions (Hong et al., 2014). Left forward lunge produced significantly higher first and second peaks of vertical impacts compared to backward lunges, and higher maximum anterior-posterior shear forces compared to left backward lunges (Hong et al., 2014). Backhand forward lunges were also related to larger trunk rotation, greater demand in core control and dynamic postural stability compared to forehand forward lunges (Lin et al., 2015). However, compared to backward lunge, Chen et al. (2020) reported that forward lunge produced higher compressional ankle contact, faster touchdown hip abduction, and larger horizontal deceleration of the mass center and torso. Among forward lunges, participants performing in-front forward lunge experienced significantly smaller load on hallux but a larger load on the lateral midfoot (Hu et al., 2015). However, it should be noted that performing lunge in a single movement would be biomechanically different from that of the repeated movements. Participants performing repeated lunge demonstrated shorter foot contact time and smaller impact, peak knee anteroposterior force, peak knee sagittal movement, but larger peak horizontal GRF (Lam, Ding & Qu, 2017). The differences in movement strategies may allow for more consistent execution of consecutive ballistic movements (Cormack et al., 2008).

Among the three lunge variations (kick, step-in, and hop), step-in lunge was considered as a more energy-saving/efficient move, since involvement of the non-lunging (non-racket side) leg aided in support and reduced GRF of the leading foot (Kuntze, Mansfield & Sellers, 2010). It also generated less hip joint power and, thus, a smaller contribution to the recovery phase (Kuntze, Mansfield & Sellers, 2010). In contrast, hop lunge produced significantly higher joint power output for quicker movement, as indicated by larger GRF during loading response and drive-off phases, larger peak ankle moment, and ankle and knee joint powers (Kuntze, Mansfield & Sellers, 2010). The greater plantarflexor power was associated with improved mechanical efficiency by enhancing the muscular stretch-shortening cycle and subsequently dissipating energy to recover back to the initial position quickly (Kuntze, Mansfield & Sellers, 2010).

## Methodological Quality Assessment

With respect to the assessment of methodological quality, six out of the 20 reviewed articles (30%) scored less than 50%. Most of the articles failed to indicate their source of population and sampling method clearly that may impose selection bias and affected the external validity. While blinding was impossible because participants were asked to perform different tasks, the majority of the articles implemented randomized crossover trials to avoid carry-over effects. Tables 1 and 2 shows that there were differences in the definitions of higher-skill players and instructions to conduct the lunge, which may contribute to the heterogeneity and variance amongst the results of the reviewed articles. In addition, there were disparities in the variable definition. For example, Lam, Ding & Qu (2017) defined the approaching distance from the initial position to the lunging footstrike, whereas Kuntze, Mansfield & Sellers (2010) calculated the approaching speed by the distance including the returning move.

## Discussion

A lunge is fundamental footwork that characterizes by extremely large footstrike angles and extraneous movements. It is primarily determined by explosive strength (high power output at high velocity) but compromised by agility required to facilitate change of direction, acceleration/deceleration, and hence quick returns (Phomsoupha & Laffaye, 2015). Scientific studies in lunges have not been well established in badminton (Chen et al., 2017; Cronin, McNair & Marshall, 2003; Fu, Ren & Baker, 2017), but all included articles agreed that the lunge step is related to performance and risks of injury in the lower limb. A good lunge performance is paramount to better upper limb racket control (Lees, 2003) and performance in competition. However, most of the reviewed articles utilized a step-in lunge protocol with maximum exertion, which may not adequately and pragmatically render the conditions of the game. Kuntze, Mansfield & Sellers (2010) evaluated and compared three other types of lunge styles (kick, step-in, and hop), which may require a performance analysis to address the frequency of different styles.

Skilled players would generate high velocity and deceptive strokes (Maloney, 2018), which was demonstrated by a substantially large knee joint moment (Fu, Ren & Baker, 2017; Lam et al., 2017; Mei et al., 2017). Specific kinematics and kinetics studies at knee have allowed comprehensive analysis on how professional athletes use joint coordination to accommodate explosive force. In contrast, some other articles suggested that athletes with knee pain followed a more conservative knee motion and inferior weight-shifting capacity (Huang et al., 2014; Lin et al., 2015).

**Figure 2:**
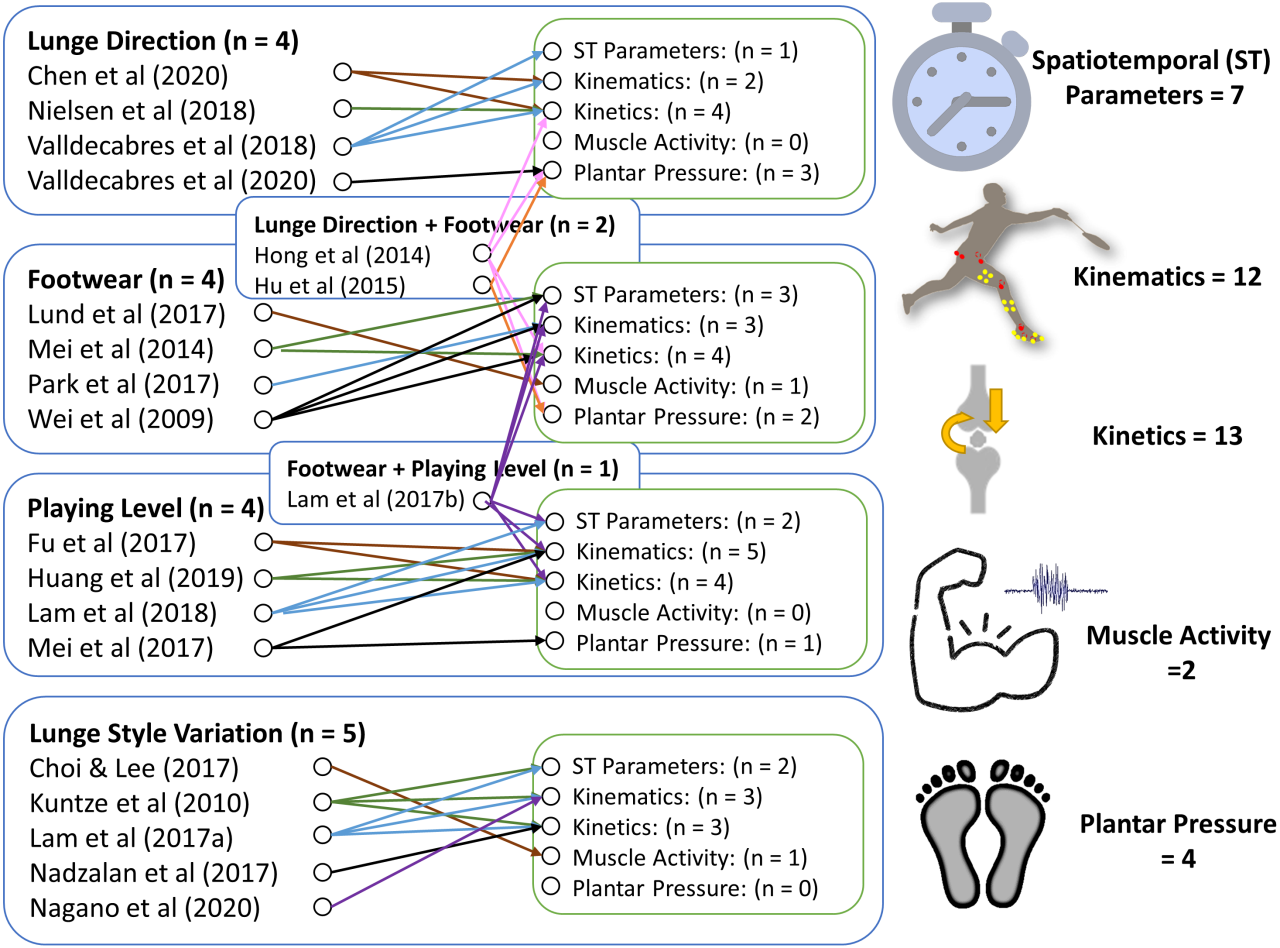
Mapping of variants and biomechanical evidence of the review articles.

Meanwhile, the articles investigating spatiotemporal parameters, GRF, and muscle activity have provided key information on how different strategies affect the strength quality of the lunge and the risk of injury. Footwear stiffness and comfort could contribute to the agility and muscle work performance (Luo et al., 2009; Nigg, 2001; Park et al., 2017) indicated by plantar pressure profiles. Figure 2 shows a map that provided a summary of the variants being associated with certain biomechanics. Professional athletes can produce higher power and utilize muscle force efficiently by reducing redundancy in force generation through joint coordination (Phomsoupha & Laffaye, 2015).

Badminton injuries ranked the sixth amongst all sports injuries, and nearly half of the badminton injuries involved the lower extremity (Phomsoupha & Laffaye, 2020). Muscle fatigue was believed to be a crucial factor, although only one article investigated proprioceptive knee brace during fatigue and provided some evidence on the use of knee orthosis (Valldecabres et al., 2018) in our review. Muscle fatigue impairs the muscle coordination and exaggerates the stress or tension on other relative vulnerable and highly loaded soft tissue such as the Achilles tendon and the patella tendon (Phomsoupha & Laffaye, 2020). The impact during lunge represents another source of injury, particularly during the intensive lunge in which the Achilles tendon and the patella tendon are loaded six to 12 times, and five times body weight, respectively (Lee & Yoo, 2012). Compared to amateur athletes, a lower incidence of injury was found among professional athletes, which could be explained by smaller peak horizontal GRF and loading rates (Herbaut, Delannoy & Foissac, 2018; Lam et al., 2017). Through the adaption of different techniques and preferred movement paths, athletes quickly adjusted movement mechanics to dissipate stresses and accommodate impact and other abrupt conditions (Huang et al., 2014; Lam, Ding & Qu, 2017; Lin et al., 2016). Moreover, the forehand and backhand forward lunges were two critical lunge directions with greater foot impact loading (Hong et al., 2014; Hu et al., 2015), in addition to the higher frequency in the game (Valldecabres et al., 2020). The backhand forward lunge could be more challenging since the players may be in a trunk position with less core stability and knee dynamic stability (Lee & Loh, 2019).

Footwear could alter movement control and/or improve sports performances. External GRF impacts could be reduced by adequate attenuation with material and structural designs of the sports shoes (Hong et al., 2014; Li, Yu & Tsai, 2018). While softer or thicker material was suggested to absorb shock, better impact attenuation remained inconclusive among badminton lunge studies (Lin et al., 2016; Lund et al., 2017; Mei et al., 2014; Park et al., 2017) even though the participants gave better comfort perception (Park et al., 2017). In fact, optimizing both shock absorption and proprioception is an important issue that needs to be resolved (Chen et al., 2017). Reduction in shock transmission can prevent proximal joint injuries, at the cost of reduced sensory information that impairs agility and balance (Lafortune & Hennig, 1992; Lin et al., 2016). Optimizing both material and structural designs could require further investigations (Lam et al., 2017; Wei et al., 2009).

The current review identified some knowledge gaps that should address in the future. No included articles compared both playing levels and lunge directions simultaneously, which was necessary to identify the biomechanical performance of all lunge directions in higher-skilled players. In fact, all research comparing playing levels only evaluated the lunge in forward forehand (right) direction. Furthermore, articles comparing lunge directions suggested more investigations on backward and backhand directions that may manifest challenges to athletes on trunk rotation and dynamic stability (Lin et al., 2015). While a single investigation addressing all playing levels and lunge directions may not be reproducible due to the variety of study scope, experimental protocols and variables across different articles should be unified to minimize heterogeneity for meta-analysis. On the other hand, research on footwear often targeted impact attenuation that is related to the impact-related injury. There was a lack of investigation to demonstrate improved performance, such as approaching speed or dexterity in different footwear modifications.

We identified some conflicting evidence in this review. Higher-level athletes may or may not produce greater knee and ankle joint angles and moments. While the variation could be due to the differences in lunge directions or variations, instructions may influence the players’ decisions to prioritize lunging speed and thus larger mechanical output, otherwise, speed to return position and thus less mechanical output. For example, some included articles limited recovery time of the lunge period (Lam et al., 2018) whilst some included articles required the players to achieve maximum (Lam, Ding & Qu, 2017; Lam et al., 2017) or pre-set lunge distance (Hu et al., 2015). Moreover, Lam, Ding & Qu (2017) argued that an isolated lunge test using a controlled setting was far from the reality of interest in a competition. The relationship between isolated lunge, repeated lunge (Lam, Ding & Qu, 2017), or uncontrolled lunge during a badminton game (Nagano et al., 2020) required further investigations.

There were some limitations to this review. Some related articles (Hong et al., 2016; Phomsoupha, Berger & Laffaye, 2018), such as those with agility and change of direction tests, were not included as they did not present any biomechanical parameters on the lunge movements. In addition, the current review did not include articles that aimed at the relationship between biomechanical attributes and injuries. We found two excluded articles that compared the biomechanics of lunge between players with and without knee pain (Huang et al., 2014; Lin et al., 2015). However, it does not necessarily mean that lower-level badminton athletes are exposed to biomechanical conditions of the knee pain athletes. Our review found that higher-level players tended to be more aggressive (higher joint moment) and thus susceptible to injury. A more focused review should identify their critical points during lunge to improve strength training.

Future studies can consider other badminton footwork/manoeuvres, including side and crossover stepping and overhead-smash landing (Kimura et al., 2011; Kuntze, Sellers & Mansfield, 2009). Moreover, a cross-sectional review across different kinds of sports (e.g., fencing) with lunge can also be performed to quantify sport-specific variations and requirements (Chen et al., 2017; Wong, Lee & Lam, 2020). Regarding the risk of injury, little attention was paid to ankle biomechanics such as loading at the Achilles tendon, which is related to badminton injury (Fahlström, Björnstig & Lorentzon, 1998; Kaalund et al., 1989). To understand the underlying mechanism of this injury better, the application of EMG, ultrasound imaging techniques, and near-infrared spectroscopy are necessary to monitor the changes in cross-section area and activity during lunges for muscles and other soft tissue (Chen et al., 2019a; Guo et al., 2010). While muscle fatigue could implicate the risk of injury, a variability analysis on inter-limb coordination is useful to evaluate the instability induced by muscle fatigue (Robertson et al., 2013; Hamill et al., 1999), despite the findings from some articles that the compensatory walking mechanisms against fatigue may interfere the analysis (Wong, Lam & Lee, 2020; Wong et al., 2015). In addition, muscle fatigue could be measured by near-infrared spectroscopy studying the hemodynamic changes (Tan et al., 2020). On the other hand, computational simulation using finite element analysis can provide a versatile platform to understand the underlying mechanism of isolated risk factors interplayed with footwear constructions related to impact and soft tissue responses (Chen et al., 2019b; Wong et al., 2016; Yu et al., 2016).

## Conclusions

This review study provided key findings of badminton lunge biomechanics. Joint kinematics, plantar pressure, and GRF were common parameters used to assess badminton lunge biomechanics. Professional athletes generally showed aggressive knee and ankle strategy (higher joint moment) and impact attenuation capabilities compared with the amateurs. Except for comfort perception, the majority of the studied footwear constructions did not demonstrate strong evidence about functional benefits and performance improvement. In addition, this scoping review identified research gaps, including backward and backhand lunge, the interaction between playing levels and lunge directions, as well as conflicting findings in joint kinematics, possibly due to variations in lunge and instructions.

##  Supplemental Information

10.7717/peerj.10300/supp-1 Supplemental Information 1 Results of literature search and label of excluded articles

## Additional Information and Declarations

### Data Availability

The following information was supplied regarding data availability: This is a literature review; the raw data consists of the results of the literature search and labels of excluded articles.
