# Supplementary material for: Biomechanics of lower limb in badminton lunge: a systematic scoping review
Source: PeerJ. 2020 Nov 4;8:e10300. doi: 10.7717/peerj.10300 (PMC7648456; doi:10.7717/peerj.10300)
Supplement: Supplemental Information 1 [file peerj-08-10300-s001.docx]

**Search Results of Pubmed (n = 21)**

1. Chen TL, Wang Y, Wong DW, Lam WK, and Zhang M. 2020. Joint contact force and movement deceleration among badminton forward lunges: a musculoskeletal modelling study. Sports Biomech:1-13. 10.1080/14763141.2020.1749720
2. Cronin J, McNair PJ, and Marshall RN. 2003. Lunge performance and its determinants. J Sports Sci 21:49-57. 10.1080/0264041031000070958
3. Fu L, Ren F, and Baker JS. 2017. Comparison of Joint Loading in Badminton Lunging between Professional and Amateur Badminton Players. Appl Bionics Biomech 2017:5397656. 10.1155/2017/5397656
4. Hong Y, Wang SJ, Lam WK, and Cheung JT. 2014. Kinetics of badminton lunges in four directions. J Appl Biomech 30:113-118. 10.1123/jab.2012-0151
5. Hu X, Li JX, Hong Y, and Wang L. 2015. Characteristics of Plantar Loads in Maximum Forward Lunge Tasks in Badminton. Plos One 10:e0137558. 10.1371/journal.pone.0137558
6. Huang MT, Lee HH, Lin CF, Tsai YJ, and Liao JC. 2014. How does knee pain affect trunk and knee motion during badminton forehand lunges? J Sports Sci 32:690-700. 10.1080/02640414.2013.848998
7. Huang P, Fu L, Zhang Y, Fekete G, Ren F, and Gu Y. 2019. Biomechanical Analysis Methods to Assess Professional Badminton Players' Lunge Performance. J Vis Exp. 10.3791/58842
8. Kuntze G, Mansfield N, and Sellers W. 2010. A biomechanical analysis of common lunge tasks in badminton. J Sports Sci 28:183-191. 10.1080/02640410903428533
9. Lam WK, Ding R, and Qu Y. 2017a. Ground reaction forces and knee kinetics during single and repeated badminton lunges. J Sports Sci 35:587-592. 10.1080/02640414.2016.1180420
10. Lam WK, Lee KK, Park SK, Ryue J, Yoon SH, and Ryu J. 2018. Understanding the impact loading characteristics of a badminton lunge among badminton players. Plos One 13:e0205800. 10.1371/journal.pone.0205800
11. Lam WK, Ryue J, Lee KK, Park SK, Cheung JT, and Ryu J. 2017b. Does shoe heel design influence ground reaction forces and knee moments during maximum lunges in elite and intermediate badminton players? Plos One 12:e0174604. 10.1371/journal.pone.0174604
12. Lee JJJ, and Loh WP. 2019. A state-of-the-art review on badminton lunge attributes. Comput Biol Med 108:213-222. 10.1016/j.compbiomed.2019.04.003
13. Lin CF, Hua SH, Huang MT, Lee HH, and Liao JC. 2015. Biomechanical analysis of knee and trunk in badminton players with and without knee pain during backhand diagonal lunges. J Sports Sci 33:1429-1439. 10.1080/02640414.2014.990492
14. Mei Q, Gu Y, Fu F, and Fernandez J. 2017. A biomechanical investigation of right-forward lunging step among badminton players. J Sports Sci 35:457-462. 10.1080/02640414.2016.1172723
15. Nagano Y, Sasaki S, Higashihara A, and Ichikawa H. 2020. Movements with greater trunk accelerations and their properties during badminton games. Sports Biomech 19:342-352. 10.1080/14763141.2018.1478989
16. Nielsen MH, Lund JN, Lam WK, and Kersting UG. 2020. Differences in impact characteristics, joint kinetics and measurement reliability between forehand and backhand forward badminton lunges. Sports Biomech 19:547-560. 10.1080/14763141.2018.1501086
17. Park SK, Lam WK, Yoon S, Lee KK, and Ryu J. 2017. Effects of forefoot bending stiffness of badminton shoes on agility, comfort perception and lower leg kinematics during typical badminton movements. Sports Biomech 16:374-386. 10.1080/14763141.2017.1321037
18. Phomsoupha M, and Laffaye G. 2015. The science of badminton: game characteristics, anthropometry, physiology, visual fitness and biomechanics. Sports Med 45:473-495. 10.1007/s40279-014-0287-2
19. Thijs Y, Van Tiggelen D, Willems T, De Clercq D, and Witvrouw E. 2007. Relationship between hip strength and frontal plane posture of the knee during a forward lunge. Br J Sports Med 41:723-727; discussion 727. 10.1136/bjsm.2007.037374
20. Valldecabres R, de Benito AM, Littler G, and Richards J. 2018. An exploration of the effect of proprioceptive knee bracing on biomechanics during a badminton lunge to the net, and the implications to injury mechanisms. Peerj 6:e6033. 10.7717/peerj.6033
21. Valldecabres R, Richards J, and De Benito AM. 2020. The effect of match fatigue in elite badminton players using plantar pressure measurements and the implications to injury mechanisms. Sports Biomech:1-18. 10.1080/14763141.2020.1712469

**Search Results for Scopus (n = 26)**

1. Chen TLW, Wang Y, Wong DWC, Lam WK, and Zhang M. 2020. Joint contact force and movement deceleration among badminton forward lunges: a musculoskeletal modelling study. Sports Biomechanics. 10.1080/14763141.2020.1749720
2. Cronin J, McNair PJ, and Marshall RN. 2003. Lunge performance and its determinants. Journal of Sports Sciences 21:49-57. 10.1080/0264041031000070958
3. Fu L, Ren F, and Baker JS. 2017. Comparison of Joint Loading in Badminton Lunging between Professional and Amateur Badminton Players. Applied Bionics and Biomechanics 2017. 10.1155/2017/5397656
4. Fu WJ, Wei Y, and Liu Y. 2015. Effects of footwear and barefoot on movement coordination of lower extremities and metatarsophalangeal joints during push-off in badminton footwork. Yiyong Shengwu Lixue/Journal of Medical Biomechanics 30:159-166. 10.3871/j.1004-7220.2015.02.159
5. Herbaut A, Delannoy J, and Foissac M. 2018. Injuries in French and Chinese regular badminton players. Science and Sports 33:145-151. 10.1016/j.scispo.2018.02.001
6. Hong Y, Wang SJ, Lam WK, and Cheung JTM. 2014. Kinetics of badminton lunges in four directions. Journal of Applied Biomechanics 30:113-118. 10.1123/jab.2012-0151
7. Hu X, Li JX, Hong Y, and Wang L. 2015. Characteristics of plantar loads in maximum forward lunge tasks in badminton. Plos One 10. 10.1371/journal.pone.0137558
8. Huang MT, Lee HH, Lin CF, Tsai YJ, and Liao JC. 2014. How does knee pain affect trunk and knee motion during badminton forehand lunges? Journal of Sports Sciences 32:690-700. 10.1080/02640414.2013.848998
9. Huang P, Fu L, Zhang Y, Fekete G, Ren F, and Gu Y. 2019. Biomechanical analysis methods to assess professional badminton players' lunge performance. Journal of Visualized Experiments 2019. 10.3791/58842
10. Kuntze G, Mansfield N, and Sellers W. 2010. A biomechanical analysis of common lunge tasks in badminton. Journal of Sports Sciences 28:183-191. 10.1080/02640410903428533
11. Lam WK, Ding R, and Qu Y. 2017a. Ground reaction forces and knee kinetics during single and repeated badminton lunges. Journal of Sports Sciences 35:587-592. 10.1080/02640414.2016.1180420
12. Lam WK, Lee KK, Park SK, Ryue J, Yoon SH, and Ryu J. 2018. Understanding the impact loading characteristics of a badminton lunge among badminton players. Plos One 13. 10.1371/journal.pone.0205800
13. Lam WK, Ryue J, Lee KK, Park SK, Cheung JTM, and Ryu J. 2017b. Does shoe heel design influence ground reaction forces and knee moments during maximum lunges in elite and intermediate badminton players? Plos One 12. 10.1371/journal.pone.0174604
14. Le Mansec Y, Perez J, Rouault Q, Doron J, and Jubeau M. 2020. Impaired performance of the smash stroke in badminton induced by muscle fatigue. International Journal of Sports Physiology and Performance 15:52-59. 10.1123/ijspp.2018-0697
15. Lee JJJ, and Loh WP. 2019. A state-of-the-art review on badminton lunge attributes. Computers in Biology and Medicine 108:213-222. 10.1016/j.compbiomed.2019.04.003
16. Lin CF, Hua SH, Huang MT, Lee HH, and Liao JC. 2015. Biomechanical analysis of knee and trunk in badminton players with and without knee pain during backhand diagonal lunges. Journal of Sports Sciences 33:1429-1439. 10.1080/02640414.2014.990492
17. Lin YJ, Chang CC, Lee SC, Hsu WC, Shiang TY, Liu TH, and Chen HL. 2016. Do thicker midsoles increase shock attenuation and do thin midsoles facilitate propulsion during lunge maneuvers? Footwear design for racket-sport industry. Proceedings of the IEEE International Conference on Industrial Technology. p 1578-1584.
18. Liu SZ, Hu ZG, and Zhang J. 2015. Inverse dynamics simulation on lower limb responses from badminton athlete under impact loads. Yiyong Shengwu Lixue/Journal of Medical Biomechanics 30:30-37. 10.3871/j.1004-7220.2015.01.030
19. Maloney SJ. 2018. Review of the badminton lunge and specific training considerations. Strength and Conditioning Journal 40:7-17. 10.1519/SSC.0000000000000378
20. Mei Q, Gu Y, Fu F, and Fernandez J. 2017. A biomechanical investigation of right-forward lunging step among badminton players. Journal of Sports Sciences 35:457-462. 10.1080/02640414.2016.1172723
21. Nielsen MH, Lund JN, Lam WK, and Kersting UG. 2018. Differences in impact characteristics, joint kinetics and measurement reliability between forehand and backhand forward badminton lunges. Sports Biomechanics. 10.1080/14763141.2018.1501086
22. Park SK, Lam WK, Yoon S, Lee KK, and Ryu J. 2017. Effects of forefoot bending stiffness of badminton shoes on agility, comfort perception and lower leg kinematics during typical badminton movements. Sports Biomechanics 16:374-386. 10.1080/14763141.2017.1321037
23. Phomsoupha M, and Laffaye G. 2020. Injuries in badminton: A review. Science and Sports. 10.1016/j.scispo.2020.01.002
24. Valldecabres R, De Benito AM, Littler G, and Richards J. 2018. An exploration of the effect of proprioceptive knee bracing on biomechanics during a badminton lunge to the net, and the implications to injury mechanisms. Peerj 2018. 10.7717/peerj.6033
25. Valldecabres R, Richards J, and De Benito AM. 2020. The effect of match fatigue in elite badminton players using plantar pressure measurements and the implications to injury mechanisms. Sports Biomechanics. 10.1080/14763141.2020.1712469
26. Yong W, Yu L, Minlu T, and Weijie F. 2009. Effects of different footwear on the metatarsophalangeal joint during push-off in critical badminton footwork. Journal of Medical and Biological Engineering 29:172-176.

**Search Results for SportDISC (n = 15)**

1. n.d., 2018. Corrigendum. Journal of Sports Sciences 36:962-962.
2. Couppé C, Kongsgaard M, Aagaard P, Vinther A, Boesen M, Kjær M, and Magnusson SP. 2013. Differences in tendon properties in elite badminton players with or without patellar tendinopathy. Scandinavian Journal of Medicine & Science in Sports 23:e89-e95.
3. Herbaut A, Delannoy J, and Foissac M. 2018. Injuries in French and Chinese regular badminton players. Science & Sports 33:145-151.
4. Huang M-T, Lee H-H, Lin C-F, Tsai Y-J, and Liao J-C. 2014. How does knee pain affect trunk and knee motion during badminton forehand lunges? Journal of Sports Sciences 32:690-700.
5. Kuntze G, Mansfield N, and Sellers W. 2010. A biomechanical analysis of common lunge tasks in badminton. Journal of Sports Sciences 28:183-191.
6. Lam WK, Ding R, and Qu Y. 2017. Ground reaction forces and knee kinetics during single and repeated badminton lunges. Journal of Sports Sciences 35:587-592.
7. Lees A, and Hurley C. 1995. Forces in a badminton lunge movement. In, Reilly, T (ed) et al, Science and racket sports, London, E & FN Spon, 1995, p 186-189. ;.
8. Lin C-F, Hua S-H, Huang M-T, Lee H-H, and Liao J-C. 2015. Biomechanical analysis of knee and trunk in badminton players with and without knee pain during backhand diagonal lunges. Journal of Sports Sciences 33:1429-1439.
9. Maloney SJ. 2018. Review of the Badminton Lunge and Specific Training Considerations. Strength & Conditioning Journal 40:7-17.
10. Mei Q, Gu Y, Fu F, and Fernandez J. 2017. A biomechanical investigation of right-forward lunging step among badminton players. Journal of Sports Sciences 35:457-462.
11. Park S-K, Lam W-K, Yoon S, Lee K-K, and Ryu J. 2017. Effects of forefoot bending stiffness of badminton shoes on agility, comfort perception and lower leg kinematics during typical badminton movements. Sports Biomechanics 16:374-386.
12. Youlian H, Shao Jun W, Wing Kai L, and Tak-Man Cheung J. 2014. Kinetics of Badminton Lunges in Four Directions. Journal of Applied Biomechanics 30:113-118.

**Search Results for Web of Sciences (n = 24)**

1. Fu L, Ren F, and Baker JS. 2017. Comparison of Joint Loading in Badminton Lunging between Professional and Amateur Badminton Players. Applied Bionics and Biomechanics 2017. 10.1155/2017/5397656
2. Herbaut A, Delannoy J, and Foissac M. 2018. Injuries in French and Chinese regular badminton players. Science & Sports 33:145-151. 10.1016/j.scispo.2018.02.001
3. Hong YL, Wang SJ, Lam WK, and Cheung JTM. 2014. Kinetics of Badminton Lunges in Four Directions. Journal of Applied Biomechanics 30:113-118. 10.1123/jab.2012-0151
4. Huang MT, Lee HH, Lin CF, Tsai YJ, and Liao JC. 2014. How does knee pain affect trunk and knee motion during badminton forehand lunges? Journal of Sports Sciences 32:690-700. 10.1080/02640414.2013.848998
5. Huang P, Fu L, Zhang Y, Fekete G, Ren F, and Gu YD. 2019. Biomechanical Analysis Methods to Assess Professional Badminton Players' Lunge Performance. Jove-Journal of Visualized Experiments. 10.3791/58842
6. Jie J, Lee J, and Loh WP. 2019. A state-of-the-art review on badminton lunge attributes. Computers in Biology and Medicine 108:213-222. 10.1016/j.compbiomed.2019.04.003
7. Kuntze G, Mansfield N, and Sellers W. 2010. A biomechanical analysis of common lunge tasks in badminton. Journal of Sports Sciences 28:183-191. 10.1080/02640410903428533
8. Lam WK, Ding R, and Qu Y. 2017a. Ground reaction forces and knee kinetics during single and repeated badminton lunges. Journal of Sports Sciences 35:587-592. 10.1080/02640414.2016.1180420
9. Lam WK, Lee KK, Park SK, Ryue J, Yoon SH, and Ryu J. 2018. Understanding the impact loading characteristics of a badminton lunge among badminton players. Plos One 13. 10.1371/journal.pone.0205800
10. Lam WK, Ryue J, Lee KK, Park SK, Cheung JTM, and Ryu J. 2017b. Does shoe heel design influence ground reaction forces and knee moments during maximum lunges in elite and intermediate badminton players? Plos One 12. 10.1371/journal.pone.0174604
11. Lee JJJ, Loh WP, and Ho ZW. 2019. Significance of Gender in Badminton Lunge Classification. In: Ratnam MM, ed. International Conference on Recent Advances in Industrial Engineering and Manufacturing.
12. Lin CF, Hua SH, Huang MP, Lee HH, and Liao JC. 2018. Biomechanical analysis of knee and trunk in badminton players with and without knee pain during backhand diagonal lunges (vol 33, pg 1429, 2015). Journal of Sports Sciences 36:962-962. 10.1080/02640414.2017.1351135
13. Lin CF, Hua SH, Huang MT, Lee HH, and Liao JC. 2015. Biomechanical analysis of knee and trunk in badminton players with and without knee pain during backhand diagonal lunges. Journal of Sports Sciences 33:1429-1439. 10.1080/02640414.2014.990492
14. Lin YJ, Chang CC, Lee SC, Hsu WC, Shiang TY, Liu TH, Chen HL, and Ieee. 2016. Do thicker midsoles increase shock attenuation and do thin midsoles facilitate propulsion during lunge maneuvers?
15. Madsen CM, Badault B, and Nybo L. 2018. CROSS-SECTIONAL AND LONGITUDINAL EXAMINATION OF EXERCISE CAPACITY IN ELITE YOUTH BADMINTON PLAYERS. Journal of Strength and Conditioning Research 32:1754-1761. 10.1519/jsc.0000000000002573
16. Maloney SJ. 2018. Review of the Badminton Lunge and Specific Training Considerations. Strength and Conditioning Journal 40:7-17. 10.1519/ssc.0000000000000378
17. Mei QC, Gu YD, Fu FQ, and Fernandez J. 2017. A biomechanical investigation of right-forward lunging step among badminton players. Journal of Sports Sciences 35:457-462. 10.1080/02640414.2016.1172723
18. Nadzalan AM, Mohamad NI, Low JFL, Tan K, Janep M, and Hamzah S. 2017. KINETICS ANALYSIS OF STEP AND JUMP FORWARD LUNGE AMONG BADMINTON PLAYERS. Journal of Fundamental and Applied Sciences 9:1011-1023. 10.4314/jfas.v9i6s.74
19. Nagano Y, Sasaki S, Higashihara A, and Ichikawa H. 2020. Movements with greater trunk accelerations and their properties during badminton games. Sports Biomechanics 19:342-352. 10.1080/14763141.2018.1478989
20. Park SK, Lam WK, Yoon S, Lee KK, and Ryu J. 2017. Effects of forefoot bending stiffness of badminton shoes on agility, comfort perception and lower leg kinematics during typical badminton movements. Sports Biomechanics 16:374-386. 10.1080/14763141.2017.1321037
21. Phomsoupha M, and Laffaye G. 2015. The Science of Badminton: Game Characteristics, Anthropometry, Physiology, Visual Fitness and Biomechanics. Sports Medicine 45:473-495. 10.1007/s40279-014-0287-2
22. Valldecabres R, de Benito AM, Littler G, and Richards J. 2018. An exploration of the effect of proprioceptive knee bracing on biomechanics during a badminton lunge to the net, and the implications to injury mechanisms. Peerj 6. 10.7717/peerj.6033
23. Valldecabres R, Richards J, and De Benito AM. The effect of match fatigue in elite badminton players using plantar pressure measurements and the implications to injury mechanisms. Sports Biomechanics. 10.1080/14763141.2020.1712469
24. Wei Y, Liu Y, Tian ML, and Fu WJ. 2009. Effects of Different Footwear on the Metatarsophalangeal Joint during Push-off in Critical Badminton Footwork. Journal of Medical and Biological Engineering 29:172-176.

**Eligible Articles (n = 20)**

1. Chen TL, Wang Y, Wong DW, Lam WK, and Zhang M. 2020. Joint contact force and movement deceleration among badminton forward lunges: a musculoskeletal modelling study. Sports Biomech:1-13. 10.1080/14763141.2020.1749720
2. Fu L, Ren F, and Baker JS. 2017. Comparison of Joint Loading in Badminton Lunging between Professional and Amateur Badminton Players. Applied Bionics and Biomechanics 2017. 10.1155/2017/5397656
3. Hong Y, Wang SJ, Lam WK, and Cheung JT. 2014. Kinetics of badminton lunges in four directions. J Appl Biomech 30:113-118. 10.1123/jab.2012-0151
4. Hu X, Li JX, Hong Y, and Wang L. 2015. Characteristics of plantar loads in maximum forward lunge tasks in badminton. PLoS ONE 10. 10.1371/journal.pone.0137558
5. Huang P, Fu L, Zhang Y, Fekete G, Ren F, and Gu Y. 2019. Biomechanical analysis methods to assess professional badminton players' lunge performance. Journal of Visualized Experiments 2019. 10.3791/58842
6. Kuntze G, Mansfield N, and Sellers W. 2010. A biomechanical analysis of common lunge tasks in badminton. Journal of Sports Sciences 28:183-191.
7. Lam WK, Ding R, and Qu Y. 2017a. Ground reaction forces and knee kinetics during single and repeated badminton lunges. Journal of Sports Sciences 35:587-592.
8. Lam WK, Lee KK, Park SK, Ryue J, Yoon SH, and Ryu J. 2018. Understanding the impact loading characteristics of a badminton lunge among badminton players. PLoS ONE 13. 10.1371/journal.pone.0205800
9. Lam WK, Ryue J, Lee KK, Park SK, Cheung JT, and Ryu J. 2017b. Does shoe heel design influence ground reaction forces and knee moments during maximum lunges in elite and intermediate badminton players? PLoS ONE 12:e0174604. 10.1371/journal.pone.0174604
10. Mei Q, Gu Y, Fu F, and Fernandez J. 2017. A biomechanical investigation of right-forward lunging step among badminton players. Journal of Sports Sciences 35:457-462.
11. Nagano Y, Sasaki S, Higashihara A, and Ichikawa H. 2020. Movements with greater trunk accelerations and their properties during badminton games. Sports Biomechanics 19:342-352. 10.1080/14763141.2018.1478989
12. Nielsen MH, Lund JN, Lam WK, and Kersting UG. 2020. Differences in impact characteristics, joint kinetics and measurement reliability between forehand and backhand forward badminton lunges. Sports Biomechanics 19:547-560. 10.1080/14763141.2018.1501086
13. Park SK, Lam WK, Yoon S, Lee KK, and Ryu J. 2017. Effects of forefoot bending stiffness of badminton shoes on agility, comfort perception and lower leg kinematics during typical badminton movements. Sports Biomechanics 16:374-386. 10.1080/14763141.2017.1321037
14. Valldecabres R, De Benito AM, Littler G, and Richards J. 2018. An exploration of the effect of proprioceptive knee bracing on biomechanics during a badminton lunge to the net, and the implications to injury mechanisms. PeerJ 2018. 10.7717/peerj.6033
15. Valldecabres R, Richards J, and De Benito AM. 2020. The effect of match fatigue in elite badminton players using plantar pressure measurements and the implications to injury mechanisms. Sports Biomechanics. 10.1080/14763141.2020.1712469
16. Wei Y, Liu Y, Tian ML, and Fu WJ. 2009. Effects of Different Footwear on the Metatarsophalangeal Joint during Push-off in Critical Badminton Footwork. Journal of Medical and Biological Engineering 29:172-176.

**Eligible articles from Grey Literature**

1. Choi Y, and Lee S. 2017. Changes in lower limb muscle activity based on angle of ankle abduction during lunge exercise. Journal of physical therapy science 29:1947-1949. 10.1589/jpts.29.1947
2. Lund JN, Lam WK, Nielsen MH, Qu Y, and Kersting U. 2017. The effect of insole hardness distribution on calf muscle loading and energy return during a forward badminton lunge. Footwear Science 9:S136-S137. 10.1080/19424280.2017.1314381
3. Mei Q, Zhang Y, Li J, and Rong M. 2014. Different sole hardness for badminton movement. Journal of Chemical and Pharmaceutical Research 6:632-634.
4. Nadzalan A, Mohamad N, Low J, Tan K, Janep M, and Hamzah S. 2017. Kinetics analysis of step and jump forward lunge among badminton players. Journal of Fundamental and Applied Sciences 9:1011-1023. 10.4314/jfas.v9i6s.74

**Articles excluded because of language (n = 2)**

1. Fu WJ, Wei Y, and Liu Y. 2015. Effects of footwear and barefoot on movement coordination of lower extremities and metatarsophalangeal joints during push-off in badminton footwork. Yiyong Shengwu Lixue/Journal of Medical Biomechanics 30:159-166. 10.3871/j.1004-7220.2015.02.159
2. Liu SZ, Hu ZG, and Zhang J. 2015. Inverse dynamics simulation on lower limb responses from badminton athlete under impact loads. Yiyong Shengwu Lixue/Journal of Medical Biomechanics 30:30-37. 10.3871/j.1004-7220.2015.01.030

**Articles excluded because of article types (e.g. review) (n = 6)**

1. n.d., 2018. Corrigendum. Journal of Sports Sciences 36:962-962.
2. Jie J, Lee J, and Loh WP. 2019. A state-of-the-art review on badminton lunge attributes. Computers in Biology and Medicine 108:213-222. 10.1016/j.compbiomed.2019.04.003
3. Lin YJ, Chang CC, Lee SC, Hsu WC, Shiang TY, Liu TH, and Chen HL. 2016. Do thicker midsoles increase shock attenuation and do thin midsoles facilitate propulsion during lunge maneuvers? Footwear design for racket-sport industry. Proceedings of the IEEE International Conference on Industrial Technology. p 1578-1584.
4. Maloney SJ. 2018. Review of the Badminton Lunge and Specific Training Considerations. Strength & Conditioning Journal 40:7-17.
5. Phomsoupha M, and Laffaye G. 2015. The Science of Badminton: Game Characteristics, Anthropometry, Physiology, Visual Fitness and Biomechanics. Sports Medicine 45:473-495. 10.1007/s40279-014-0287-2
6. Phomsoupha M, and Laffaye G. 2020. Injuries in badminton: A review. Science and Sports 35:189-199. 10.1016/j.scispo.2020.01.002

**Articles excluded because they were not focusing on lunge manuevres (n = 5)**

1. Cronin J, McNair PJ, and Marshall RN. 2003. Lunge performance and its determinants. Journal of Sports Sciences 21:49-57. 10.1080/0264041031000070958
2. Herbaut A, Delannoy J, and Foissac M. 2018. Injuries in French and Chinese regular badminton players. Science & Sports 33:145-151.
3. Le Mansec Y, Perez J, Rouault Q, Doron J, and Jubeau M. 2020. Impaired performance of the smash stroke in badminton induced by muscle fatigue. International Journal of Sports Physiology and Performance 15:52-59. 10.1123/ijspp.2018-0697
4. Madsen CM, Badault B, and Nybo L. 2018. Cross-Sectional and Longitudinal Examination of Exercise Capacity in Elite Youth Badminton Players. Journal of Strength and Conditioning Research 32:1754-1761. 10.1519/JSC.0000000000002573
5. Thijs Y, Van Tiggelen D, Willems T, De Clercq D, and Witvrouw E. 2007. Relationship between hip strength and frontal plane posture of the knee during a forward lunge. Br J Sports Med 41:723-727; discussion 727. 10.1136/bjsm.2007.037374

**Articles excluded because they recruited unhealthy participants (pain, or pathological) (n = 3)**

1. Couppé C, Kongsgaard M, Aagaard P, Vinther A, Boesen M, Kjær M, and Magnusson SP. 2013. Differences in tendon properties in elite badminton players with or without patellar tendinopathy. Scandinavian Journal of Medicine & Science in Sports 23:e89-e95.
2. Huang MT, Lee HH, Lin CF, Tsai YJ, and Liao JC. 2014. How does knee pain affect trunk and knee motion during badminton forehand lunges? Journal of Sports Sciences 32:690-700. 10.1080/02640414.2013.848998
3. Lin CF, Hua SH, Huang MT, Lee HH, and Liao JC. 2015. Biomechanical analysis of knee and trunk in badminton players with and without knee pain during backhand diagonal lunges. Journal of Sports Sciences 33:1429-1439. 10.1080/02640414.2014.990492
